# New Findings on the Biology and Ecology of the Ecuadorian Amazon Fungus *Polyporus leprieurii* var. *yasuniensis*

**DOI:** 10.3390/jof8020203

**Published:** 2022-02-20

**Authors:** Cristina E. Toapanta-Alban, María E. Ordoñez, Robert A. Blanchette

**Affiliations:** 1 Department of Plant Pathology, University of Minnesota, St. Paul, MN 55108, USA; robertb@umn.edu; 2 Fungarium QCAM, Escuela de Ciencias Biológicas, Facultad de Ciencias Exactas y Naturales, Pontificia Universidad Católica del Ecuador, Quito 170143, Ecuador; meordonez92399@gmail.com

**Keywords:** Basidiomycota, competition studies, fungi, macroecology, melanin, microcosms, rhizomorphs, tropical rainforest, Yasuní National Park, wood decay

## Abstract

*Polyporus leprieurii* var*. yasuniensis* is a prolific wood-decay fungus inhabiting the forest floor of one of the most biodiverse places on earth, the Yasuní National Park in Ecuador. Basidiocarps and aerial rhizomorphs are commonly found growing on woody debris distributed along the floor of this forest ecosystem. Because of the extraordinary abundance of this fungus in the tropical rainforest, we carried out investigations to better understand the biological and ecological aspects contributing to its prolific distribution. Data on growth inhibition in paired competition studies with sixteen fungal isolates exemplifies defense mechanisms used to defend its territory, including pseudosclerotial plates and the development of a melanized rhizomorphic mat. Results of biomass loss on eleven types of tropical wood in microcosm experiments demonstrated the broad decay capacity of the fungus. In and ex situ observations provided information on how long rhizomorphs can prevail in highly competitive ecosystems as well as stressful conditions in the laboratory. Finally, high concentrations of metal ions occur on rhizomorphs as compared to colonized wood. Sequestration of metal ions from the environment by the melanized rhizomorphs may offer protection against competitors. The development of melanized rhizomorphs is key to find and colonize new substrates and resist changing environmental conditions.

## 1. Introduction

Studies of biological diversity provide a better understanding of the ecosystem composition, how ecosystems function, and how organisms interact with other organisms in their surrounding environment. This leads to a deeper understanding of the life history of organisms: lifespan/longevity, relative abundance (rarity/commonness) and the biological traits that make them unique as they function in the ecosystem. This information also contributes to conservation assessments for species and communities.

In ecology, a general observation is that most species are rare, and few species are common [1,2,3]. This is especially true in tropical ecosystems, which are known for holding most of the known biodiversity on the planet [4,5,6,7], for instance Yasuní National Park, the biggest natural reserve in Ecuador, is known for its high species richness for amphibians, birds, mammals, and vascular plants [8]. However, its fungal species richness is still not well documented. A preliminary study to understand the species richness of poroid wood decay fungi in Yasuní showed that most species of polypores were infrequently found, and a few were common, following the previously mentioned species richness pattern [9]. Most recent surveys in the area suggest that species within the *Polyporus* s.l. of the Polyporaceae family were among the most common taxa found in this ecosystem, and most of them are closely related to *Polyporus leprieurii* Mont. [10].

*Polyporus leprieurii* Mont. was described and proposed by Camille Montagne in 1840 while describing other taxa from French Guiana [11]. Initially, its distribution range was established in tropical to subtropical regions of America and Eastern Asia [11,12]. New records are commonly based on morphological descriptions matching Montagne [11] and Núñez and Ryvarden’ [12] descriptions. Such macro- and micromorphological descriptions in terms of pilear shape and color, stipe position and color, hyphal system, and spore shape and size can be observed across its known synonyms, including *P. hemicapnodes* Berk. & Broome, *P. pusillus* Rostr., *P. calyculus* Pat., & Gaillard, *P. savoyanus* Pat., Revue, *P. subelegans* Murrill, *P. tephromelas* Mont., and *P. atripes* Rostr. [13]. The phylogenetic relationships between the “true” *P. leprieurii* and its known synonyms are still unresolved, and a recent study suggested that *P. leprieurii* is a species complex that needs further revision to resolve its true taxonomy [10]. In similar studies, findings have revealed other taxa within the “Melanopus group”, and new genera have been proposed [14,15,16]. Therefore, until the true phylogenetic relationships are resolved in the taxa sharing morphological similarities with *P. leprieurii*, we refer to these taxa in a broad sense as a part of the “leprieurii” group [10].

**Figure 1:**
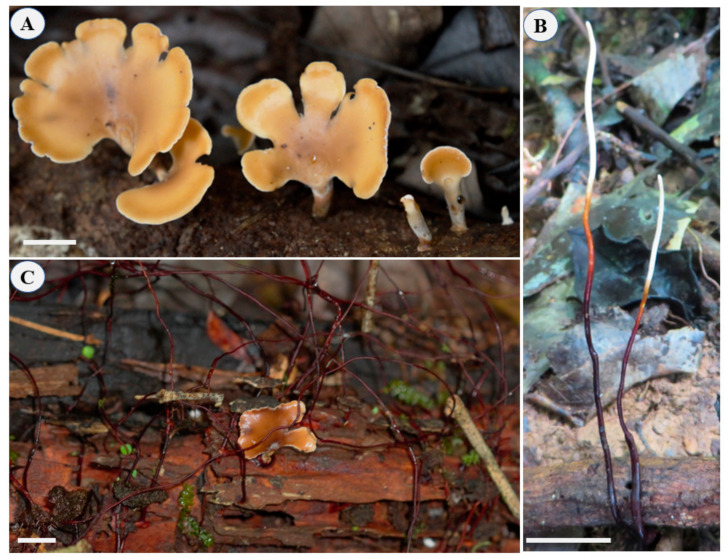
*Polyporus leprieurii* var. *yasuniensis*. (**A**) Primordia and fresh basidiocarps. (**B**) Aerial rhizomorphs. (**C**) Complex network of rhizomorphs accompanying basidiocarps on wood substrate. Scale bars = 1 cm.

*Polyporus. leprieurii* var. *yasuniensis* (Figure 1), the taxon used in this research, is a new variety proposed by Toapanta-Alban et al., as micromorphological and genetic variations were found to be present between freshly collected samples from Yasuní and herbarium samples and DNA sequences from Belize and Costa Rica [10].

In terms of abundance among the *Polyporus*-like specimens found in Yasuní, *P. leprieurii* var. *yasuniensis* is the most common taxon along the forest floor, with numerous collection sites marked and surveyed in the Yasuní forest dynamic permanent plot [10,17]. This variety is often found growing on both fine-woody debris (FWD) and coarse woody debris (CWD), where basidiocarps and rhizomorphs are easily found together on the same substrate (Figure 1C) [10]. However, rhizomorphs are more abundant than basidiocarps. In this taxon, rhizomorphs can be single to highly ramified cord-like structures bearing a dark outer layer of melanized tissue (Figure 1B,C). They undergo anastomosis to form complex networks emerging from woody debris and grow out to attach to small plants and other vegetative material to maintain an upward growth pattern [10].

In polypores, rhizomorph formation is a strategy used to respond to changes in their environment and to search for new substrates to colonize [12]. In temperate forests, rhizomorphs have been observed to be key in the pathogenicity of *Armillaria* species complex [18,19,20,21,22,23,24,25]. *Armillaria* species produce rhizomorphs below ground and can also be found under the bark of infected trees [18,19,20,21,22,23,24,25]. *Polyporus leprieurii* and allied taxa are different and produce their rhizomorphs above ground [10]. The biological significance of *Armillaria* rhizomorphs has received considerable research attention [24,25,26,27,28,29]. However, in tropical ecosystems, information on the biological significance and ecological functions of above ground rhizomorphs is limited. A few studies have focused on species forming aerial rhizomorphs, usually found in the canopy of trees, and their association with birds that use them as nesting material [30,31,32,33]. In addition, Isaac et al. described that saprophytic fungi with the ability to form rhizomorphs are likely to capture resources faster than the fungi with no rhizomorph production [34]. These non-unit-restricting fungi grow from one substrate to another, often colonizing nutrient-deficient litter by translocating nutrients from other decaying resources [34].

Basic research on the biology and ecology of tropical fungi-producing rhizomorphs is needed, especially in Yasuní National Park. It is important to fill this gap in our knowledge due to the importance of wood-decay fungi in nutrient cycling and their other functions in forest ecosystems [35,36,37] and to better understand the advantages of fungi producing rhizomorphs in niche colonization.

In this research paper, we present information on several biological traits of *P. leprieurii* var. *yasuniensis*, including its response to interactions with other wood-inhabiting fungi in dual competition studies, its decay capacity on tropical woods and its affinity for melanized tissue to absorb metal ions from the environment. In addition, we discuss ecological aspects on longevity and persistence of rhizomorphs in and ex situ. This information provides a better understanding about the role this fungus plays in tropical forests and provides important new information on the biology and ecology of this fungus and potentially other similar taxa present in tropical ecosystems worldwide.

## 2. Materials and Methods

This study was carried out in the 50 ha Permanent Plot at the Yasuní National Park, Ecuador (0°41′0.5″ S 76°23′58.9″ W) [17] between December 2016–December 2019 and with laboratory studies. Research was carried out under a QCAM Fungarium research permit of the Pontificia Universidad Católica del Ecuador. Additional permits were issued by the Environmental Ministry of Ecuador in Francisco de Orellana to transport collections to the QCAM Fungarium in Quito, where samples were accessioned.

Basidiocarps and rhizomorphs of the fungi used here were sampled using a random stratified sampling method (ten quadrants of 100 × 100 m in areas with no trail and six transects of 100 × 20 m along the main trails) to cover a significant proportion of the plot that included all topographical habitats [10]: ridges, slopes and valleys (terra firme and temporary flooded forest) as described in Valencia et al. [38] and to determine the abundance and distribution along the plot. Fungal isolates and fungal identifications were based on taxonomic observations (macro and microscopic features) and molecular identification following the protocols in Toapanta-Alban et al. and Blanchette et al. respectively [10,39].

### 2.1. Competition Studies

Cultures of *P. leprieurii* var. *yasuniensis* (CT-16F) were paired with other fungal isolates obtained from decayed wood, fresh basidiocarps, senescent basidiocarps and rhizomorphs of *P. leprieurii* var. *yasuniensis* found at the field location in Yasuní National Park (Table 1).

In vitro dual competition studies and controls (CT-16F alone) were established using two types of media, malt extract agar (MEA) and malt yeast agar (MYA). Media were prepared following Toapanta-Alban et al. [10]. Assays were established as follows. In plastic Petri dishes (90 × 15 mm) containing 20 mL of agar media, 5 mm disks from two-week old cultures of each fungus were placed 5 cm apart and stored at room temperature (20–21 °C) for 28 weeks. Five plates were used for each combination and media type. After three days, growth was recorded daily for a week for the detection of fast-growing fungi and then weekly for 8 weeks, followed by monthly readings to check for viability of mycelium until 28 weeks. At ten weeks, inhibition growth was calculated as follows: ((R–r)/R) × 100, where R is the radial growth in the control, and r is the radial growth when paired with the other fungi [40,41]. Additional notes describing the development of rhizomorphs and the interaction at the mycelial level (gross-mycelial contact) were noted following Woodward and Boddy [42] and Doty observations [43]. Such notes include pigmentation of superficial mycelium (melanization), zone-lines (pseudosclerotial plates), overgrowth, aerial tufts, mycelial cords, and replacement (relic zone lines).

The success of *P. leprieurii* var. *yasuniensis* (CT-16F isolate) or the other fungi was determined by the inhibition growth at 10 weeks and the mycelial response at 28 weeks. In the case that opposite responses were observed among repetitions, success was determined by the majority. Conversely, if there was not a clear sign of which fungus dominated the growth (“winner”), success was noted as equal.

### 2.2. Decay Study

**Figure 2:**
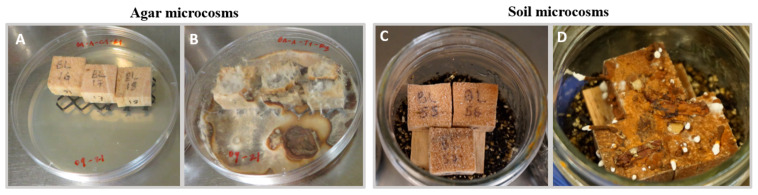
Agar and soil microcosms. Non-inoculated (**A**,**C**) and inoculated blocks after 30 days (**B**,**D**).

Soil and agar microcosms were used to test the capacity of *P. leprieurii* var. *yasuniensis* for wood degradation (Figure 2).

Decay studies were carried out using *P. leprieurii* var. *yasuniensis* CT-16F isolate on wood blocks from eleven tropical woods with different densities (Table 2) [44].

Wood was obtained at local sawmills in Quito and Quevedo (Ecuador). Wood was cut into 2 × 2 × 2 cm^3^ woodblocks, except for laurel that was cut into 1.5 × 1.5 × 1.5 cm^3^ woodblocks due to the amount of available wood. Each woodblock was labeled and then oven-dried at 100 °C for 48 h, cooled down in a desiccation chamber, and individual weight was recorded as w1. Blocks were then submerged in deionized water for 24 h for rehydration and autoclaved twice in glass Petri dishes (containing 8 blocks max) for 60 min cycles at 121 °C. Once cooled, blocks were promptly placed in the microcosms.

For soil microcosms, soil mixture was prepared with two parts of soil, two parts of vermiculite, and one part of peat moss thoroughly mixed to remove rocks and big pieces of organic matter. Soil mixture was placed in 16 oz (wide mouth) mason jars using a 50 mL beaker, then 45 mL of deionized water was carefully added, and two wood feeder-strips (1.5 cm wide × 3 cm length) were placed on top of the soil. Jars with the soil mixture, water, and feeder strips were autoclaved twice for 60 min at 121 °C. Once cooled, 5 mm disks of inoculum from two-week-old cultures were placed on each corner of the feeder strips and allowed to grow for 4 weeks. For controls, mock inoculation was performed.

Agar microcosms were modified from the methods of Schilling and Jacobson [45] using MYA. Agar microcosms were set up using plastic Petri dishes (10 × 2.5 cm) with 15 mL of MYA and a piece of plastic mesh (previously autoclaved for two 60 min cycles at 121 °C) 7 × 2.5 cm on top of agar. Three 5 mm disks of inoculum were placed one cm apart on the other extreme of the mesh and left to grow for two weeks. For controls, no inoculum was placed in microcosms. In both the agar and soil microcosms, three wood blocks were placed in each Petri-dish/jar with one block harvested at 30, 60, and 90 days. Five replications of each wood type were used. All microcosms were incubated at room temperature (20–21 °C) inside plastic containers.

When woodblocks were harvested, mycelium was carefully removed from the surface of blocks and then oven-dried at 100 °C for 48 h. Blocks were cooled in a desiccation chamber, and the weight of each block was recorded as w2. Mass loss was calculated as follows: ((w1 − w2)/w1) × 100, where w1 is initial weight and w2 is final weight. Results are expressed as an average of the percentage of weight loss for each type of wood, incubation time, and microcosm type. At the end of the experiments, soil microcosms that had produced rhizomorphs on feeder strips were kept for longevity and survival tests.

### 2.3. Longevity and Survival

Longevity and survival of rhizomorphs was observed and tested in and ex situ. In situ observations were gathered over a period of three years to check for the viability and presence of rhizomorphs on collection sites marked as highly dense. Ex situ observations were gathered in soil microcosms with rhizomorphs produced in the laboratory.

#### 2.3.1. In Situ

One hundred nine collection sites resulted from the random stratified sampling method focused on *Polyporus*-like species. One hundred of these were associated with rhizomorphs’ production. *Polyporus leprieurii* var. *yasuniensis* was present in forty-six collection sites, demonstrating its high local abundance compared to other taxa producing rhizomorphs, *P. taromenane* (3 collection sites), and *A. yasuniensis* (1 collection site) [10]. Local abundance is defined by the number of individuals living at one time in a given area [46,47]. Since the term “individuals” can be difficult to apply to fungi with clonal (vegetative) organs as rhizomorphs [48], we considered each collection site, which is at least 20 m apart from the next one, as one individual. In this scenario, each rhizomorph colonizing one substrate was considered as one genet [49]. We did not focus on counting genets on each collection site, as this work was extenuating due to their abundance.

The area occupied in each collection site/individual of *P. leprieurii* var. *yasuniensis* varied from 0.5 to 40 m^2^. Due to a large amount of fine and coarse woody debris colonized by rhizomorphs and basidiocarps, we decided to focus on the number of living plants colonized by rhizomorphs at each collection site; these varied from zero to thirty-one. With this information, and due to a large number of collection sites, we selected the collection sites/individuals with an occupied area greater than 5 m^2^ for the long-term surveys and focused on changes on the colonized area and number of living plants colonized. We also checked for viability of rhizomorphs and basidiocarps. Another parameter used to select these areas was accessibility, as some of the collection sites were difficult to revisit due to the number of fallen trees, flooded terrain, and wasp nests.

**Figure 3:**
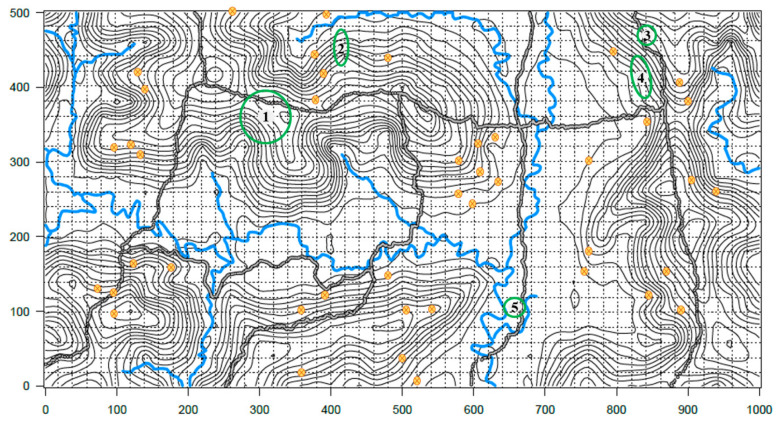
Yasuní Forest Dynamic Plot, 50 ha topographical map showing collection sites. Yellow circles correspond to collection sites not included in longevity observations. Areas in green correspond to collection sites included in longevity observations. **1**. CT-16F, **2**. CTR-2-12, **3**. CTR-2-27, **4**. CTR-2-31, and **5**. CTR-2-67. Elevation lines correspond to 5 m changes. Choppy grid equivalates to 20 m^2^. Grey and black lines mark the trails. Blue lines correspond to streams. This map was created by the Forest dynamic laboratory, Pontificia Universidad Católica del Ecuador, and it was modified to show our collection sites.

The occupied area was delimited with a fixed-area plot sampling method [48] that we adjusted for fungi. First, the biggest and/or highest colonized substrate was marked as the center of the collection site. Then, with the measuring tape, the area was surveyed for more colonized substrates at every meter (at each cardinal point) until no colonized substrates were found. In December 2016, five collection sites were selected and marked for long-term observations: CT-16F (previously found in November 2015), CTR-2-12, CTR-2-27, CTR-2-31, and CTR-2-67 (Figure 3).

Sites were found in all topographical habitats, including, ridges, slopes, and valleys (terra firme and temporary flooded areas). In each site, the occupied area and the number of colonized plants were noted at the beginning and end of surveys. In December 2019, the last visit was carried out to check for the presence of viable rhizomorphs in all sites.

#### 2.3.2. Ex Situ

**Figure 4:**
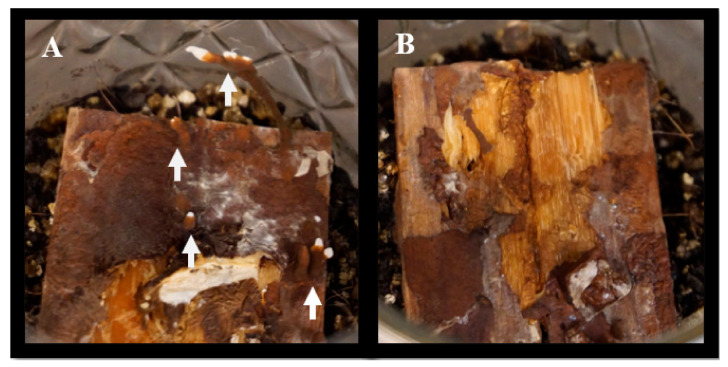
Soil microcosms at the end of the decay study (120 days old) after all woodblocks were harvested. (**A**) Feeder strips with rhizomorphs. Arrows show rhizomorphs. (**B**) Feeder strips without rhizomorphs.

Ex situ survival was assessed with rhizomorphs produced in soil microcosms during the decay study (Figure 4).

Production of rhizomorphs ex situ was previously observed to occur on old MYA cultures [10]. However, it is still unclear what the exact parameters were that led to the development of rhizomorphs under laboratory conditions. Therefore, we decided to take advantage of the rhizomorphs produced on feeder strips during the decay study and expose rhizomorphs to different conditions to measure their survival. The variables that we decided to control were light and water, considering that in situ rhizomorphs are naturally exposed to temporary droughts and floods on ridges and valleys, and variations on light exposure occur on all topographical habitats due to forest gaps.

Fifty-two jars from the decay study were selected for having rhizomorphs growing on feeder strips. Rhizomorphs varied in quantity and size in each jar; thus, jars were randomly selected to form five groups that corresponded to the following treatments (Table 3).

For two groups (1 and 2), sterile deionized water was added to each jar to simulate flooded conditions. No water was added to the three remaining groups. Light exposure was suppressed to one group with water and another without water, by keeping jars inside a black box (1 and 3). One group with water and one with no water had light exposure by keeping jars in clear plastic containers (2 and 4). Groups 1, 2, 3, and 4 were placed on a metal shelf near a window. For group 5, light and moisture content remained unchanged by keeping jars in the same place they were during the wood decay study (with no direct sunlight exposure).

In each jar, the number of rhizomorphs was recorded at the beginning and end of the experiment. At the end of the experiment (450 days since microcosms were established), rhizomorph viability was assessed by identifying actively growing tissue (white apical tips on rhizomorphs). When no active growing tissue was identified, viability was confirmed by isolating rhizomorphs and/or a segment of mycelial mat on MYA. Jars that failed to have active growing tissue and were not successfully isolated were marked as dead.

### 2.4. Elemental Analysis

Multi-elemental analysis for Al, B, Ba, Be, Ca, Cd, Co, Cr, Cu, Fe, K, Li, Mg, Mn, Mo, Na, Ni, P, Pb, Rb, S, Si, Sr, Ti, V, and Zn was determined in dried rhizomorphs and their wood substrates. This was performed using Inductively Coupled Argon Plasma Spectrometry (ICP-AES) dry-ash method at the Research Analytical Laboratory, University of Minnesota. Rhizomorphs and wood substrates from two sites were thoroughly rinsed with deionized water to remove soil particles and then dried at 50 °C. Then, 500 mg of each sample were used in the analysis with methods previously described in Held et al. [28]. Results are presented in mg/kg for each element in samples.

## 3. Results

### 3.1. Competition Studies

In vitro dual competition studies served to observe the inhibition growth of *P. leprieurii* var. *yasuniensis* when combined with other fungi, as well as to identify mycelial responses upon interaction.

**Figure 5:**
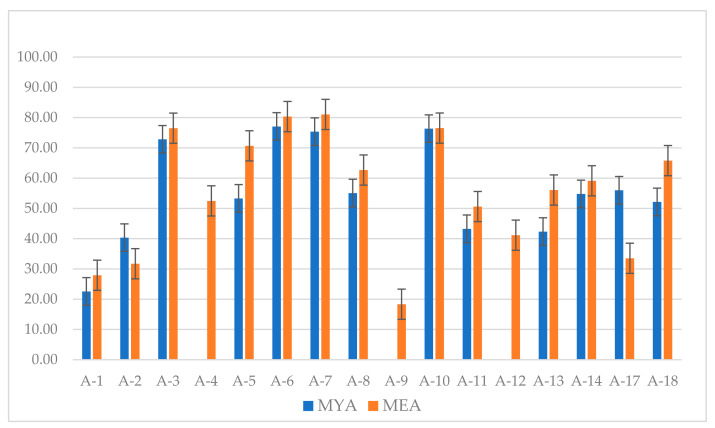
Inhibition growth of *P. leprieurii* var. *yasuniensis* after 10 weeks of growth in dual competition studies with antagonistic fungi A-1 to A-18. Results are shown as percentage values for both MYA and MEA with their respective bar errors.

At ten weeks, greater radial growth of *P. leprieurii* var. *yasuniensis* resulted in less inhibition growth (Table S1). On MYA, inhibition growth below 50% was reported in A1, A2, A4, A9, A11, A12, A13, and A15; while on MEA, A1, A2, A9, A11, A12, and A17. Greatest inhibition above 75% was found for A6, A7, and A10 on MYA and A3, A6, A7 and A10 on MEA. Conversely, no inhibition resulted at 10 weeks for A4, A9, and A12 on MYA (Figure 5) (Table S1).

**Figure 6:**
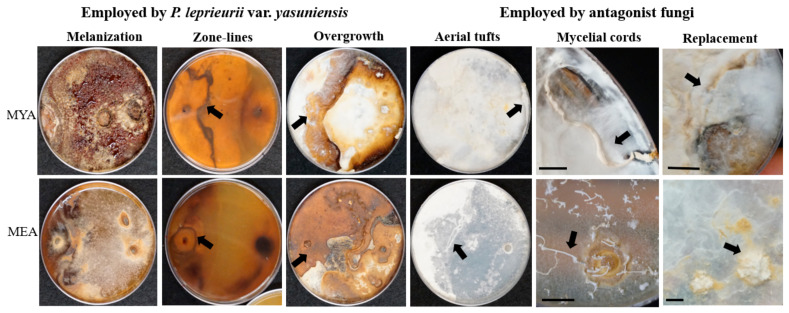
Responses observed at 28 weeks on MYA and MEA when *P. leprieurii* var. *yasuniensis* was paired with various other fungi. All antagonist fungi were place on the left side of the Petri dish. Melanization (A1); zone-lines (A13) back of the plate; overgrowth (A8); aerial tufts (A6); mycelial cords (A14); replacement with relic zone-lines (A3). Arrows point to response. Scale bar 1 cm.

By the end of the study (28 weeks), mycelial contact was confirmed on all combinations (Table S2). All responses, melanization of mycelium (a), zone lines (b), overgrowth (c), aerial tufts (d), mycelial cords (e), and replacement (d) were confirmed with visual observations (Figure 6).

**Figure 7:**
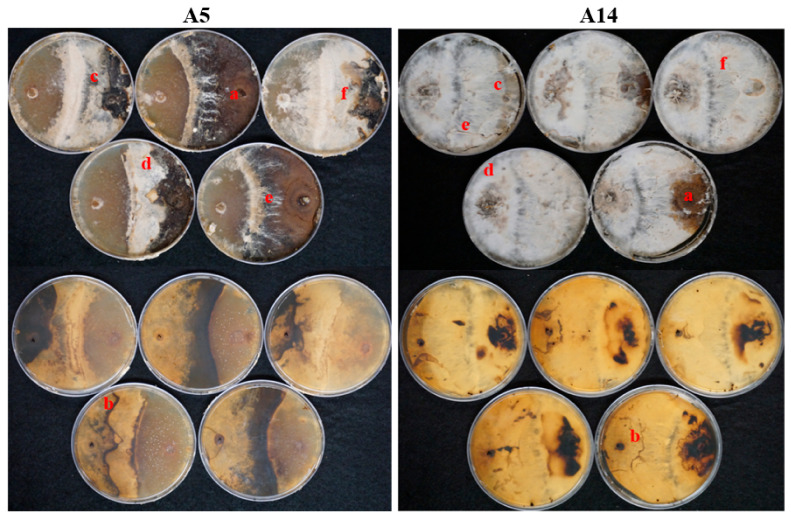
All mycelial gross contact responses observed on MYA by the end of the experiment on A5 (**left**) and A14 (**right**). *P. leprieurii* var. *yasuniensis* plug is on the right on all Petri dishes. The top five pictures correspond to the front, and the five bottom correspond to the back of Petri plates for a better observation of zone lines. Letters placed on each response. **a**. melanization; **b**. zone-lines; **c**. overgrowth; **d**. aerial tufts; **e**. mycelial cords; and **f.** replacement.

Melanization of mycelium was the response most observed, which was absent only on A6 and A7 (MYA) and A3, A6, and A14 (MEA). This response was determined when light to dark brown-reddish superficial pigmentation was present in the mycelium. The development of mycelial cords, conversely, was the response least observed and was only found on A5 and A14 (MYA) and A12 and A14 (MEA). Furthermore, combinations A5 and A14 (MYA) had evidence of all responses. In these combinations, the antagonist was noted as successful due to the percentage of inhibition growth, 62% and 81%, respectively and mycelial responses, including the development of mycelial cords. (Figure 7).

Rhizomorphs can develop on MYA cultures after six months. However, the development of rhizomorphs was not observed on any of the combinations with paired fungi. Success, when a fungus was noted as “winner”, was observed on seven combinations on MYA and six on MEA for *P. leprieurii* var. *yasuniensis*, while antagonists were successful “winners” on five combinations on MYA and six combinations on MEA (Table S2). In five combinations for both MYA and MEA, success of either *P. leprieurii* var. *yasuniensis* or the antagonist was not concluded due to number of repetitions favoring one or the other or because radial growth seemed to be equal and both organisms delimitated their territory well. This was observed on A8, A10, A13, A17, and A18 (MYA) and A11, A12, A13, A17, and A18 (MEA). In A4, A8, A10, A11, and A12, response and result of interaction changed upon media type with *P. leprieurii* var. *yasuniensis* being more successful on MYA and the antagonist being more successful on MEA (Table S2).

### 3.2. Decay Study

**Figure 8:**
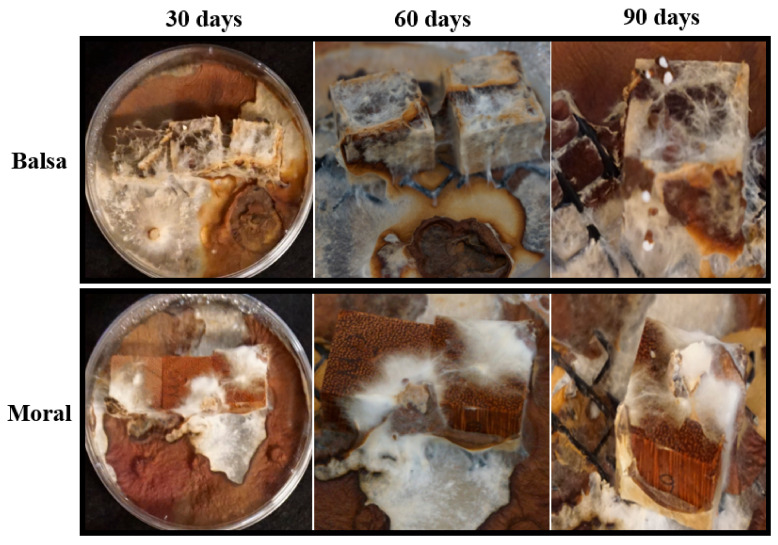
Woodblocks in agar microcosms showing colonization and rhizomorph development on balsa (the wood with the greatest decay) and moral (the wood with the lowest amount of decay) at 30, 60, and 90 days.

**Figure 9:**
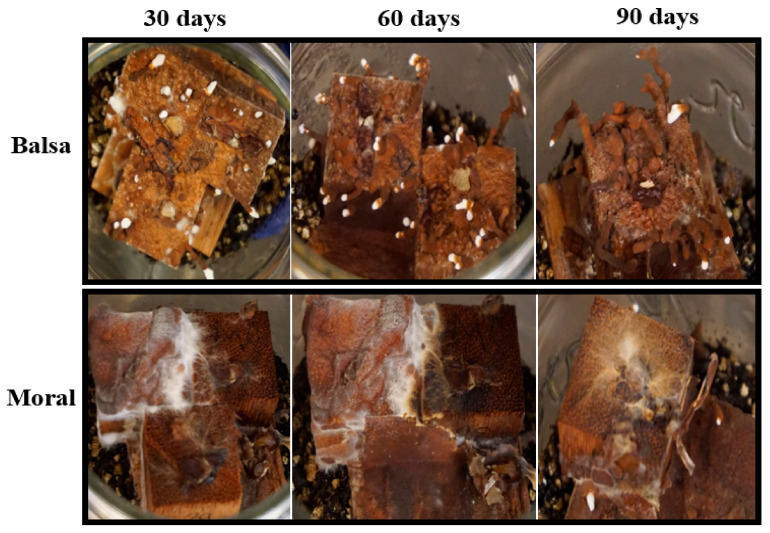
Woodblocks in soil microcosms showing colonization and rhizomorph development on balsa (the wood with the greatest decay) and moral (the wood with the lowest amount of decay) at 30, 60, and 90 days.

Wood decay studies were established to test the capacity of *P. leprieurii* var. *yasuniensis* to colonize and decompose wood from different tree species and with different wood densities. Fungal colonization on woodblocks was more substantial in soil microcosms than in agar microcosms (Figure 8 and Figure 9) (Table S3).

This was characterized by white, floccose silky initial mycelium that turned into thick, crustose to coriaceous with light to dark brown-reddish hues. As a result of the colonization of woodblocks, rhizomorph development was always observed in soil microcosms, except for on moral wood (30 and 60 days), and was only present on balsa wood on agar microcosms at 90 days (Figure 8 and Figure 9) (Table S3).

**Figure 10:**
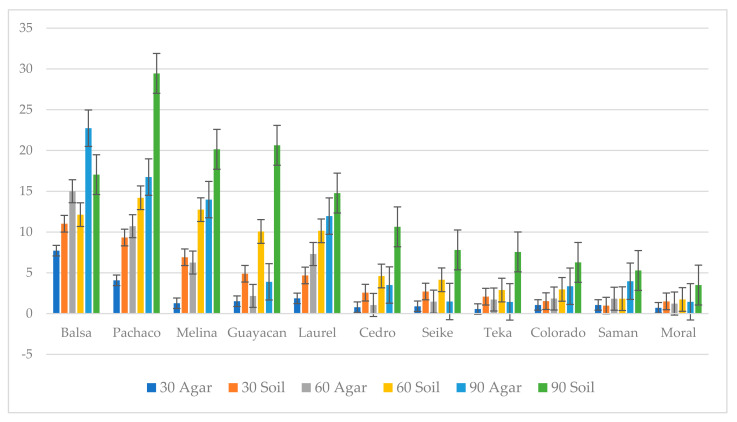
Percentage weight loss on agar and soil microcosms at 30, 60 and 90 days for all wood types.

Non-inoculated woodblocks in both agar and soil microcosms had little to no weight loss (0% to 1.30% with an average of 0.14% and standard deviation of 0.132) in controls after 30, 60, and 90 days (Table S4). In treatments with the fungus, decay progressively increased over time in all wood types. Wood with high density exhibited less decay than wood with low density. Differences in decay caused by *P. leprieurii* var. *yasuniensis* occurred among the different wood types, with balsa, pachaco, melina, guayacan, and laurel woods having greater weight loss and cedro, seike, teka, Colorado, saman, and moral woods having lower weight loss (less than 10%) (Figure 10) (Table S5).

### 3.3. Longevity and Survival

In and ex situ observations demonstrated that rhizomorphs are structures able to persist for relatively long periods under challenging environmental conditions.

#### 3.3.1. In Situ Longevity

In situ observations served to detect patterns of growth and dispersal of rhizomorphs within a period of 3 (CTR-2-12, CRE-2-27, CTR-2-31, and CTR-2-67) and 4 years (CT-16F) in highly abundant collection sites. Changes were reported in meters to see if the colonized area increased or decreased and by counting living plants colonized by rhizomorphs at the beginning (December 2016) and end of surveys (July 2019). One collection site decreased in colonized area (CT-16F), two increased in area (CTR-2-27 and CTR-2-67) and the last two maintained the same area (CTR-2-12 and CTR-2-31) (Table 4).

**Figure 11:**
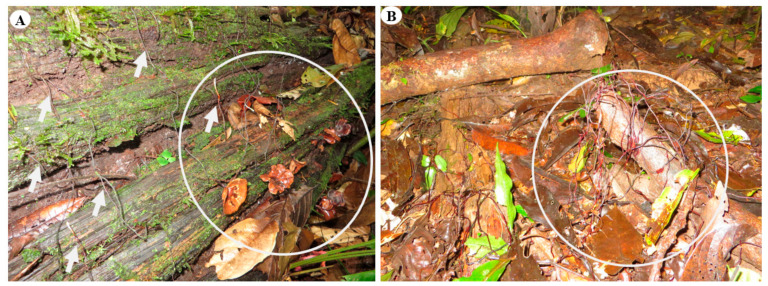
Basidiocarps and rhizomorphs found at collection sites after three years. (**A**) CT-16F collection site, fallen “mother tree” (*Plinia* sp.) colonized by basidiocarps and rhizomorphs. (**B**) CTR-2-67 collection site with fresh rhizomorphs emerging from coarse-woody debris that grew out to colonize other logs. White circle and arrows show rhizomorphs and basidiocarps on substrate.

In all sites, at the end of surveys, survival was confirmed with fresh basidiocarps and rhizomorphs emerging from old and new substrates (coarse and fine-woody debris). In these densely colonized sites, considerable amounts of coarse and fine-woody debris and dead trees were found fully colonized, except in CTR-2-67, where only coarse and fine-woody debris were present (Figure 11).

**Figure 12:**
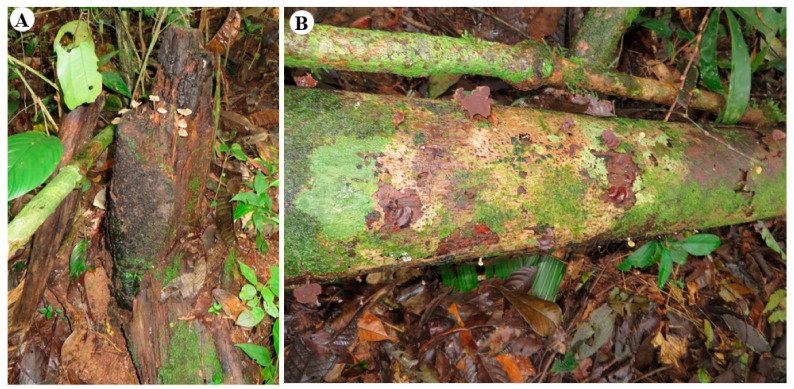
CTR-2-31, “mother tree” (*Pseudolmedia rigida*). (**A**) Symptoms of butt rot on the base of a tree. (**B**) Main trunk with basidiocarps emerging from it.

In this research, we use the term “mother tree” to refer to a whole dead tree either standing or fallen that has a tag number and exhibits the most colonization in each site. For this term, we identify the substrate that appears to be the source of dispersal of rhizomorphs and spores (emerging from basidiocarps) that will serve to colonize new substrates available in each site. “Mother trees” were identified in all sites, except for CTR-2-67 (Table S6). “Mother trees” had symptoms of butt rot and white rot, as they were broken at the root collar, and had rhizomorphs and basidiocarps present from the base to the top of trees (Figure 12).

Colonization of living trees by rhizomorphs was registered at all sites; however, with CTR-2-27 and CTR-2-31, rhizomorphs were observed to remain attached to trees since the first observation. These rhizomorphs stayed anchored to an individual substrate. In some cases, rhizomorphs remained active, while in others, rhizomorphs were attacked by insects and antagonistic fungi.

#### 3.3.2. Ex Situ Survival

Laboratory investigations allowed us to expose rhizomorphs to different conditions, simulating flooding and droughts with direct, undirect and no light exposure. By the end of the observations, rhizomorphs survived and were viable for at least 330 days in 41 of the 52 jars (Table 5).

**Figure 13:**
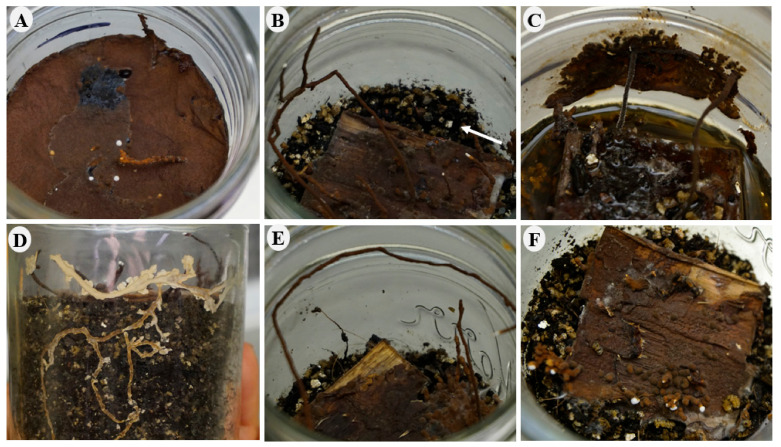
Responses of rhizomorphs and mycelium in soil microcosms. (**A**) Development of melanized mycelial mat above water. (**B**) Growth oriented up toward the light. (**C**) Growth of melanized mycelial mat and rhizomorphs on glass wall. (**D**) Above- and below-ground development of rhizomorphs. (**E**) Extensive growth of rhizomorphs. (**F**) Primordial development of rhizomorphs on wood and soil surface.

The number of rhizomorphs tended to increase for most treatments except in group 2. In all treatments, a melanized mycelial mat was observed covering the substrate (feeder strips) and spread above the soil. Mycelium was found to regrow over older tissue, developing white mycelium that eventually became melanized. Other responses observed in the mycelial mat and rhizomorphs included: the development of a melanized mycelial mat above the water in flooded treatments; upward-oriented growth of rhizomorphs in all jars with no light; attachment of mycelial mat and rhizomorphs on the glass wall; below-ground growth of rhizomorphs; extensive growth of rhizomorphs; and constant primordial development of rhizomorphs (Table 5) (Figure 13).

By the end of the experiments (450 days after microcosms were set up), rhizomorph viability was 79% for all treatments (Table 5). Most mortality (60%) was reported in treatment 4 (no water and no light), while most survival (100%) was observed in treatment 3 (no water added and with direct light exposure). Survival in treatment 5 (controls) was 80%; however, rhizomorphs did not grow as extensively as observed in the other treatments.

### 3.4. Elemental Analysis

Multi-element analysis of rhizomorphs and wood substrates of *P. leprieurii* var. *yasuniensis* (two rhizomorphs and two wood substrates) revealed higher concentrations of metal ions on rhizomorphs than in their wood substrates (Table 6).

Metals with the highest concentrations were Ca (3450 and 9707), K (1708 and 1437), Mg (809 and 1333), Al (668 and 297), Fe (399.5 and 90), and Mn (100 and 56). For these metals, concentrations in rhizomorphs were 5-, 5-, 1-, 12-, 15-, and 4-fold times more than in their wood substrate, respectively. The metals with the lowest concentrations were Rb (7.98 and 5.927), Ni (3.88 and 0.950), Ti (3 and 0.747), Cr (1.48 and 0.520), V (1.40 and 0.356), Pb (0.74 and 0.235), Co (0.47 and 0.087), Li (0.24 and 0.100), Mo (0.099), and Be (0.09). These elements were also higher in rhizomorphs than in wood substrates. Only Ni had similar concentrations in rhizomorphs as in the wood substrate (Table 6).

## 4. Discussion

*Polyporus leprieurii* var. *yasuniensis* is widespread along all topographical habitats in Yasuní National Park, where it exhibits relatively high local abundance compared to other taxa within the “Melanopus” group [10]. In this research, we focused on understanding how this fungus behaves and what allows it to exhibit high local abundance and to prevail in such a highly diverse and highly competitive ecosystem, such as Yasuní National Park. We were able to observe how *P. leprieurii* var. *yasuniensis* interacts with other fungi in controlled environments, assess its ability to decay tropical woods with different densities, report the longevity of rhizomorphs in and ex situ, and determine the content of metal ions in rhizomorphs and their wood substrates. More importantly, we were also able to observe its dispersal methods and growth patterns in densely colonized sites in the field.

### 4.1. Competition Studies

Among wood-decay fungi, competition for territory has been reported as the most common type of interaction [50]. The competition reported here can be considered as primary, because both fungi are colonizing an uncolonized substrate [42,50]. In nature, uncolonized substrates can be found when fresh limbs or entire trees fall due to abiotic factors. These are colonized by fungi that will compete to become established in the newly available substrate [42]. To better understand how these interactions between fungi occur in nature, in vitro competition studies offer a way to observe the response of fungi to competition [40,41,42,50,51,52]. In dual-competition studies, fungal responses can be detected at the mycelial level, where competitors exhibit mechanisms to gain and defend territory. In our experiments, *P. leprieurii* var. *yasuniensis* displayed three main responses to gain and protect terrain and prevent inhibition, these were overgrowth, melanization of superficial mycelium, and formation of pseudosclerotial plates. The level of responses during the interaction with the paired fungi varied as *P. leprieurii* var. *yasuniensis* was more efficient against other wood-decay fungi such as *Marasmius* sp. (A1, A2, A11), *Tinctoporellus* sp. (A8, A10), and *Ganoderma* sp. (A12). Fast growth melanization of superficial mycelium, overgrowth, and the use of pseudosclerotial plates were key to gain and delimit its territory. However, with some other wood-decay fungi such as *Caripia montagnei* (A13)*, Ganoderma weberianum* (A18)*,* and *Cristataspora coffeata* (A18), the inhibition growth and final response were established as equal since both fungi exhibited responses that prevented the competitor of invading the other’s territory by exhibiting similar growth rates and the formation of pseudosclerotial plates and a thick layer of mycelium where contact occurred. Conversely, the inhibition and responses of *P. leprieurii* var. *yasuniensis* when paired with fungi isolated from senescent rhizomorphs and those considered plant pathogens, such as Russulales (A3), Ascomycota 1 and 3 (A5, A14), *Rhizoctonia* sp. (A6), and Ascomycota 2 (A7) resulted in higher inhibition of growth and absence of defense responses that allowed the antagonist to dominate the territory. These plant pathogens and saprophytic fungi demonstrated faster growth to overgrow *P. leprieurii* var. *yasuniensis*. Samples A5 and A14 even developed mycelial cords on top of *P. leprieurii* var. *yasuniensis.* This response was also observed on the substrate where A14 was isolated, as two fungi were isolated from a piece of wood hosting, *Caripia montagnei* (A13) and Ascomycota. 3 (A14). During this experiment, rhizomorphs did not develop during the interaction with antagonistic fungi, we believe this could be the result of the competition as the fungus has to direct more energy to defense mechanisms rather than to nutrient exploration.

In this competition assay, we decided to use two media types, MYA and MEA, because the growth of *P. leprieurii* var. *yasuniensis* was reported to be favored by the yeast extract in MYA, where eventually rhizomorphs can be produced [10]. Thus, we wanted to observe its response in media that favors its growth and in another type of media where it grows slower. In MYA, yeast extract provides nitrogen, vitamins, and other growth nutrients [53], while the malt extract offers high carbohydrate content for rapid growth [54]. The inhibition of growth by *P. leprieurii* var. *yasuniensis* to competitors was somewhat higher in MEA (where its growth is slower than in MYA) for all combinations except when paired with *Cristataspora coffeata* (A17), where it was slightly higher in MYA. For five combinations (A4, A8, A10, A11, and A12), the “winner” of the interaction was reported as opposite upon the media type, while *P. leprieurii* var. *yasuniensis* was successful on MYA, and on MEA the antagonist-resulted “winner” of the interactions. These results suggest that more studies should be carried out to understand how the type of media and nutrient availability can influence competition among these tropical fungi. This could indicate a method of niche specialization among wood-decay fungi.

In our study, inhibition of growth was the result of defense mechanisms exemplifying mycelial gross contact responses that were confirmed by the changes in the morphology of mycelium when contact occurred between the paired fungi. Mycelial gross contact responses are common in dual competition studies [40,41,42,43]. However, in other in-vitro competition studies, inhibition of growth can be the result of an interaction lacking mycelial contact between the paired fungi [41]. This phenomenon is known as antibiosis and it has been documented in dual competition studies with *Trichoderma* spp. [41,52]. In our assays, none of the interactions resulted in growth inhibition caused by antibiosis, and all defense mechanisms were detected at the mycelial level after contact occurred. It is possible that *Trichoderma* would be effective at inhibiting the growth of *P*. *leprieurii* var. *yasuniensis*, as the genus is widely used in the control of plant pathogens [40,41,52,55], however, this should be tested to understand how *P. leprieurii* var. *yasuniensis* responds to antibiosis and the role of the melanized tissue in defense mechanisms against compounds secreted by *Trichoderma*.

Some of the antagonistic fungi were not able to be identified on the genus level because their DNA sequences did not have good matches to well-identified sequences available on NCBI. These fungi have not received much research attention and may represent new species. In addition, for the three taxa Ascomycota 1, 2, and 3 (CTR-3-11, CTR-3-11A, and CTR-3-26B), GenBank accession numbers were not obtained because only partial sequences were obtained for these fungi. However, we could give them some identity and infer their ecology based on the source of isolation, morphology of ascocarps, and other matches with fungal sequences in NCBI. In any case, our main objective was to identify the response of *P. leprieurii* var. *yasuniensis* when paired with fungi with similar lifestyles as well as those with different lifestyles. Future studies could take a broader approach to understand its place in wood-decay fungal communities. Another focus could be on identifying the fungi attacking *P. leprieurii* var. *yasuniensis*, to predict future threats when fungal communities change. Since this was an exploratory study, more work needs to be conducted to better understand how this fungus interacts with other taxa.

### 4.2. Wood-Decay Study

Saprophytic fungi associated with wood degradation play key roles in the decomposition of lignified organic matter and thus have important influences on the global recycling of carbon and other elements including N, P, K, S and minerals. These wood-degrading fungi make those substances available for other organisms such as plants and other fungi to utilize, which is key for healthy ecosystem functioning [36,56]. Decay studies help identify decay mechanisms employed by fungi [57,58,59] and decay rates among wood types and fungal species [59,60]. These can provide information on carbon sequestration statistics [51,61].

This study took place in Yasuní National Park, where the diversity of tree species has been reported to be extraordinarily high [8,38,62] as well as in other regions in the Amazon basin [63]. Thus, we used eleven types of tropical woods with different densities to assess decay rates when the wood is colonized by *P. leprieurii* var. *yasuniensis* to exemplify the different types of substrates the fungi available to colonize and obtain nutrients.

In both microcosm types used, most degradation was observed on balsa, pachaco, melina, and guayacan woods, which have the lowest reported heart-wood densities [44], and the lowest decay was observed in wood types with the highest densities, saman and moral wood. There are multiple reasons to explain why some wood types experienced more decay than others besides their density, for example, the extractive content in each wood type. Extractives have been reported to contribute to the longevity and durability of wood [64]. However, due to the incomplete knowledge of extractives in native Ecuadorean trees, we cannot make an assessment of this because information on extractives for all wood types used in this study is not available.

From the information obtained in this research, we can conclude that *P. leprieurii* var. *yasuniensis* is effective at colonizing different substrates, but the decay process seems to be slow, as most decay (30%) was observed at 90 days on balsa, the least dense type of wood. In nature, wood decay is a process involving insects, fungi, and other microorganisms [51,65,66]. In this study, we show decay rates in a controlled environment knowing that decay rates will vary in the field, where the quality of substrates in terms of decay stage and size are varied (fresh fallen trees, logs, coarse and woody debris) and widely available for colonization.

*P. leprieurii* var. *yasuniensis* colonizes wood by forming a melanized rhizomorphic mat that covers the substrate. This seems to be an effective strategy to protect the substrate from other competitors and to ensure that the wood and its nutrients are utilized. This was observed by the amount of decay in woodblocks that was completely covered by the fungus versus the ones that were not totally covered (Figure 8 and Figure 9). A similar situation has been observed with another cord-forming basidiomycete, *Phanerochaete velutina,* where most decay was observed on substrates, with most hyphal colonization on smaller pieces of wood [67]. We were also able to observe decay rates between different microcosms. Weight loss was reported to be higher in soil microcosms than in agar microcosms, where total coverage of woodblocks was reported, and rhizomorph formation was more likely to be expressed on feeder strips and wood blocks. It is possible that the agar offers a relatively easier substrate to colonize and to easily obtain nutrients; thus, the fungus is able to obtain nutrients faster than from the woodblocks.

A rapid formation of rhizomorphic melanized mats and pseudosclerotial plates around the perimeter of the substrate facilitates substrate colonization by *P. leprieurii* var. *yasuniensis* and prevents competitors from colonizing the resource. The rhizomorphic mat and pseudosclerotial plates appear to be key survival structures, while rhizomorph formation follows to grow out, explore, and colonize new substrates.

This was a preliminary study to better understand the role of *P. leprieurii* var*. yasuniensis* in the nutrient cycling in this forest ecosystem, due to its abundance and the varied substrates it has been found growing on. More research, however, needs to be conducted to identify the primary enzymes involved in wood degradation in tropical rainforests and to compare decay rates between *P. leprieurii* var. *yasuniensis* and other wood-decay fungi present at the site. In addition, it is important to understand its role in succession among wood-decay fungal communities. Some of this future work could be carried out in and ex situ to have a better understanding of its wood-decay capabilities and its role in carbon sequestration based on decay rates in the field.

### 4.3. Longevity and Survival

*Armillaria* species, reported to be the largest and oldest fungal organism on earth [29,68,69] and *Polyporus leprieurii* var. *yasuniensis* appear to share an important strategy to find and colonize substrates for nutrient acquisition, the production of rhizomorphs with an outer layer of protective melanin. Cord-forming Basidiomycota form vegetative propagules (rhizomorphs and mycelial cords) from networks of anastomosing hyphae when encountering and colonizing new substrates. These structures aid the fungus as it moves across the forest floor and as more complex networks are being formed [29,70,71,72]. These networks of rhizomorphs can extend several meters, as observed in *P. leprieurii* var. *yasuniensis* (on the highly abundant sites) [10], and even kilometers, as reported in *Armillaria* [29,68,69], to translocate nutrients to different parts of the mycelium [71,72].

To learn about some aspects of the lifestyle of *P. leprieurii* var. *yasuniensis*, especially its longevity and resistance, we studied the prevalence of rhizomorphs and basidiocarps in the field and tested their survival in soil microcosms. Our observations confirmed that similar to *Armillaria* [73], rhizomorphs of *P. leprieurii* var. *yasuniensis* are long-lasting structures capable of persisting under challenging environmental conditions such as in excess or limited moisture. In the field, a continuous production of rhizomorphs was observed for over 4 years of observations at the collection site, CT-16F. In 2015, its “mother tree” was standing with basidiocarps and rhizomorphs only present on the root collar. In 2016, this tree fell, resulting in abundant inoculum around the ridge near the base of the tree and around the main stem downhill, with fine- and coarse-woody debris colonized with abundant rhizomorphs and basidiocarps. Further distribution was the result of the colonized wood being dragged downhill by the rain, where new substrates were then colonized. This pattern of distribution was also observed on CTR-2-12, CTR-2-27, and CTR-2-31 collection sites, where “mother trees” fell downhill, dispersing significant amounts of inoculum.

In the collection sites located on terra firme (CT-16F, CTR-2-12, CTR-2-27, and CTR-2-31), rhizomorphs are confronted with seasonal droughts near forest gaps during the dry season, and there is direct exposure to UV light. Fungal melanin has been reported to offer protection against UV exposure as melanin absorbs UV radiation and transforms the energy into harmless amounts of heat [70,73,74,75,76,77,78]. It has been discussed that melanin could also offer adaptation to stressful conditions, including low temperatures, low water content, starvation, and increased radioactivity [74,75,76,77,78]. We believe that the melanin on rhizomorphs plays an important role in the survival of rhizomorphs exposed to direct sunlight with limited water access. We were able to test this in the laboratory in soil microcosms where water was not added but had light exposure (group 4). By the end of the experiment, rhizomorphs’ survival in group 4 was 60%, where soil and substrate were significantly dry. In the other groups, where water and light were absent (group 3) and the control (group 5) had no water and partial light, survival was 100% and 80%, respectively. Survival was relatively high considering the time rhizomorphs have been growing under those conditions with no other source of carbon but the small wood feeder strips.

Conversely, we observed rhizomorph prevalence under seasonal flooding in the field (CTR-2-67 collection) and in the laboratory in soil microcosms where water was added (groups 1 and 2). In the field, inoculated wood with rhizomorphs and basidiocarps were found underwater with rhizomorphs maintaining their upward growth to stay above the water level. By the end of the surveys in the field, their prevalence and occupied area confirmed that rhizomorphs were constantly developing at this site despite seasonal flooding. In the laboratory assay, survival in groups 1 and 2 was 82% and 73%, respectively. With water covering the soil and substrate, a melanized rhizomorphic mat was key to keep the fungus alive under these conditions. Melanized mycelial mats provide protection to the fungus in temporary flooded environments. This tissue will eventually develop rhizomorphs seeking to colonize new substrates and escape the harsh flooded conditions. Melanin is a hydrophobic pigment with high molecular weight compounds [74,75,76,77,78], a quality useful in overcoming desiccation, which may also be a strategy to escape floods by preventing excess water entering the substrate.

Rhizomorphs are key to help the fungus find new substrates and move from one substrate to another [12,19,21,25,34,70,71,72]. *P. leprieurii* var. *yasuniensis* has been shown to have a high tolerance to both desiccation and water excess with direct or absent light exposure. The development of rhizomorphs appears to prolong the longevity and prevalence of the fungus by generating asexual organs resistant to changing environmental conditions, and the melanized outer layer provides protection from attack by other organisms. More studies should be conducted to have a better estimate of the age of these individuals in each collection site, including genetic studies to identify how big these individuals are. The tropical rainforests likely harbor massive cord-forming fungi that may rival *Armillaria.*

### 4.4. Elemental Analysis

Fungal melanized tissue has been reported to play significant roles in pathogenic fungi [75,79,80,81,82,83,84], be present in extremophile fungi [29,76,82,85,86,87,88,89,90,91], accumulate heavy metals [29,92,93,94,95,96], and help fungi in a wide variety of adaptations [74,75,76,77,78,97]. The effect of melanin enhancing the survival of fungi under adverse conditions is mainly due to its function as an extracellular redox buffer, which can neutralize oxidants generated by the fungus in response to environmental stress [78]. *Polyporus leprieurii* var. *yasuniensis* exhibits dark brown to reddish pigmentation on its stipe, rhizomorphs, and mycelial mats, and it is also observed on mycelia growing in culture media as a coriaceous tissue covering the culture as well as the pseudosclerotial plates [10]. Because melanin has chelating properties [29,78,92,95], the presence of melanin in the outer layer of rhizomorphs in *P. leprieurii* var. *yasuniensis* can also absorb metal ions. Rhizomorphs exhibit much higher concentrations of metal ions as compared to the content in wood substrates. Similar results have been reported for *Armillaria* rhizomorphs [92], where Al, Zn, Fe, Cu, and Pb showed high concentrations. This phenomenon has also been observed in pseudosclerotial plates of *Phellinus weirii* exposed to soil [98]. It is also known that metals have key roles in enzyme and protein production (metalloproteins) that are likely to be key in essential metabolic pathways [99]. It is intriguing that the above ground production of rhizomorphs can sequester metal ions from the rainforest environment. With *Armillaria* and *Phellinus weirii*, contact with soil allows for high concentrations to be accumulated. In the Amazon rainforest, the aerial rhizomorphs apparently sequester metal ions from moist above-ground sources where they exist in low concentrations, but over time they can accumulate at high levels.

Some metals, such as copper, have been shown to increase the production of extracellular melanin in *Cladosporium resinae* and *Aureobasidium pullulans*, due to its role in the biosynthesis of DHN melanin polymerization [93]. The presence of melanin in fungal species offers resistance to microbial attack and survival under harsh environmental conditions [93,99]. In our study, Cu was reported to be four-fold times higher in rhizomorphs than in their wood substrate. Cu has also been reported to be 40 times higher in *Armillaria* rhizomorphs than its soil substrate [92]. The chelating property of melanin may offer resistance against microbial attack to the fungal species exhibiting melanized tissue, such as *P. leprieurii* var. *yasuniensis*. The interaction with metals and fungi has been of interest not only because metal toxicity has been used in many fungicides, but also because metals can be directly or indirectly involved in fungal growth and metabolism [100]. However, it is known that most metals will exhibit toxicity above a certain concentration [100]. We believe that the higher concentrations of metal ions on the outer layer of melanin on rhizomorphs can offer protection against antagonistic microbial taxa. Additional study is needed to better understand this process in *P. leprieurii* var. *yasuniensis*.

## 5. Conclusions

This work contributes with important new information on key biological and ecological aspects of *P. leprieurii var. yasuniensis.* This is a core species in the Amazon rainforest, where most species are rare. Its rhizomorphs help this fungus to dominate in this highly diverse ecosystem. They are efficient at finding and colonizing new substrates as well as moving out of harsh conditions (flooded areas). Melanin appears to be a key component for the success of this organism, as it provides mycelial mats and rhizomorphs with a physical barrier to prevent water loss and/or to survive excess water. It is also important as a chemical barrier when competing with other organisms for territory. The highly abundant colonized sites investigated in Yasuní National Park in this work exemplify how this organism is occupying significant areas of forest. Its growth and dispersal dynamics are extraordinary, and we speculate that this could be another massive fungal organism, present in many regions of the Amazon.

We want to encourage researchers to continue gathering biological and ecological data of rare and common fungal species to develop a better understanding of their roles in ecosystem functioning and predictions under global warming. In addition, the fungal data obtained could be included in the generation of strong conservation assessments to prioritize regions and ecological communities for fungal preservation.

## Figures and Tables

**Table 1:** List of fungi used in dual-competition studies. Brief ecological descriptions and origin of isolates, observations on melanization of cultures and GenBank accession numbers.

	Collection Number	Taxa	Ecology and Origin	Melanin in Isolates	GenBank Number
A	CT-16F	*P. leprieurii* var. *yasuniensis*	Wood-decay fungus, producing above ground rhizomorphs. Isolated from rhizomorphs.	Yes	MT950148
1	M2	*Marasmius* sp. 1	Wood-decay fungus, producing aerial rhizomorphs. Isolated from rhizomorphs.	No	OM506563
2	M4	*Marasmius* sp. 2	Wood-decay fungus, producing aerial rhizomorphs. Isolated from rhizomorphs.	No	OM506564
3	CTR-3-1	Russulales	Saprophyte fungus, isolated from senescent rhizomorphs.	No	OM506552
4	CTR-3-9	Hysterangiales	Mycorrhizae, hypogeous fungi (false-truffles). Isolated from cord-like mycelium colonizing living plants.	No	OM506553
5	CTR-3-11	Ascomycota 1	Saprophyte fungus, isolated from canker on dying palm tree.	No	*
6	CTR-3-12B	*Rhizoctonia papayae*	Plant pathogen, isolated from basidiocarps of *Rigidoporus* sp.	No	OM506554
7	CTR-3-11A	Ascomycota 2	Parasitic or plant pathogen isolated from senescent rhizomorphs.	No	*
8	CTR-3-15A	*Tinctoporellus* sp. 1	Wood-decay fungus, isolated from basidiocarps.	No	OM506555
9	CTR-3-17A	*Rhizoctonia* sp. 2	Plant pathogen, isolated from crust-like mycelium growing on leaf litter.	No	OM506556
10	CTR-3-20	*Rigidoporus microporus*	Wood-decay fungus, isolated from basidiocarps.	No	OM506557
11	CTR-3-22B	*Marasmius* sp. 3	Wood-decay fungus, isolated from sclerotia plates on decay wood.	No	OM506558
12	CTR-3-24	*Ganoderma* sp. 1	Wood-decay fungus, isolated from basidiocarps.	Yes	OM506559
13	CTR-3-26D	*Caripia montagnei*	Wood-decay, isolated from white mycelium on branches.	No	OM506560
14	CTR-3-26B	Ascomycota 3	Saprophyte, crust like fungus growing on decayed wood. It produces mycelial cords on media. Isolated from basidiocarps.	No	*
17	CTR-3-37	*Cristataspora coffeata*	Wood-decay fungus, isolated from basidiocarps.	No	OM506561
18	CTR-3-38	*Ganoderma weberianum*	Wood-decay fungus, isolated from basidiocarps.	No	OM506562

* Not available, identification made by morphological characteristics.

**Table 2:** List of wood types used in decay studies, common and scientific name, and known density of heartwood.

	Common Name	Scientific Name	Density (g/cm^3^)
1	Balsa	*Ochroma pyramidale*	0.04
2	Pachaco	*Schizolobium parahyba*	0.41
3	Melina	*Gmelina arborea*	0.58
4	Guayacan	*Tabebuia chrysantha*	0.65
5	Laurel	*Cordia alliodora*	0.38–0.64
6	Seike	*Cedrelinga cateniformis*	0.45–0.55
7	Teka	*Tectona grandis*	0.61
8	Saman	*Samanea saman*	0.72–0.88
9	Colorado	*Guarea gomma*	0.52
10	Cedro	*Cedrela odorata*	0.48
11	Moral	*Chlorophora tinctoria*	0.89

**Table 3:** Summary of treatments in ex situ assays with rhizomorphs. The number of jars used in each treatment and description of altered conditions for each treatment.

Code	Number of Jars	Treatment Conditions
1	11	100 mL of deionized water added and no light exposure.
2	11	100 mL of deionized water added and light exposure.
3	10	No water added and no light exposure.
4	10	No water added and light exposure.
5	10	No changes were made to these jars. No water was added, and jars were kept in a clear container with no direct light exposure.

**Table 4:** In situ longevity of rhizomorphs on studied sites: initial and final area colonized by rhizomorphs in radius and squared meters; initial and final number of living plants colonized by rhizomorphs; survival after three years.

	Initial Observations			After 3 Years			
Collection Site	Radius in Meters	Occupied Area m^2^	Colonized Plants	Radius in Meters	Occupied Area m^2^	Colonized Plants	Survival
* CT-16F	40	5026.54	18	35	3848	14	Yes
CTR-2-12	10 × 25	785	10	10 × 25	785	4	Yes
CTR-2-27	10	314	6	15	706.86	9	Yes
CTR-2-31	13 × 30	1225	16	13 × 30	1225	31	Yes
CTR-2-67	12	425	3	14.5	660.51	27	Yes

* CT-16F was surveyed for 4 years.

**Table 5:** Summary of the number of rhizomorphs and viability of rhizomorphs per treatment at the beginning and end of the experiment, percentage of survival, and observed mycelial mat and rhizomorphic responses.

	-Number of Rhizomorphs		Jars with Viable Rhizomorphs		% Survival of Jars	Observed Responses *
Treatment	Initial	Final	Initial	Final		
1	90	108	11	9	82	A, C, E, F
2	89	82	11	8	73	A, B, C, D, E, F
3	87	123	10	10	100	C, D, E, F
4	80	101	10	6	60	B, C, D, E, F
5	82	139	10	8	80	B, C, D, F

* A, melanized mycelial mat above water; B, oriented growth of rhizomorphs to one side of jars; C, mycelial mat and rhizomorphs attached to the glass wall; D, below-ground growth of rhizomorphs; E, extensive growth of rhizomorphs; F, primordial growth of rhizomorphs.

**Table 6:** Elemental analysis (mg/kg) of rhizomorphs and their wood substrates collected from two collection sites.

Sample	Ca *	K *	P	Mg *	Al *	Si	Fe *	S	Mn *
Rhizomorph 1	3450.4	1708.2	978.95	809.44	668.40	439.24	398.46	361.91	100
Wood 1	1235.3	288.27	205.61	664.59	10.186	26.794	16.695	323.96	25
Rhizomorph 2	9707.0	1437.0	704.01	1333.4	297.11	323.93	89.572	567.59	56.35
Wood 2	1868.5	249.16	131.88	1171.8	68.351	31.015	14.654	626.95	9.724
**Sample**	Ba *	Sr *	Na *	Zn *	Cu *	**B**	Rb *	Ni *	Ti *
Rhizomorph 1	98.248	48.737	41.752	34.269	18.651	18.084	7.981	3.883	2.989
Wood 1	69.756	100.92	7.029	4.729	5.395	3.922	<0.074	3.681	<0.003
Rhizomorph 2	41.234	64.208	218.85	42.441	10.985	6.735	5.927	0.950	0.747
Wood 2	66.939	160.74	34.983	25.682	2.620	6.676	<0.074	2.164	0.084
**Sample**	Cr *	V *	Cd *	Pb *	Co *	Li *	Mo *	Be *	
Rhizomorph 1	1.483	1.406	0.798	0.739	0.473	0.238	0.099	0.094	
Wood 1	0.189	0.020	0.175	0.414	0.049	0.058	<0.001	<0.001	
Rhizomorph 2	0.520	0.356	0.297	0.235	0.087	0.100	<0.001	<0.001	
Wood 2	0.263	0.090	0.143	0.194	<0.002	0.089	<0.001	<0.001	

* Metal elements.

## Data Availability

Data are contained within the article and Supplemental Material.

## Supplementary Materials

The following supporting information can be downloaded at: www.mdpi.com/article/10.3390/jof8020203/s1. Table S1: Radial growth in mm and percentage of inhibition growth of *P. leprieurii* var. *yasuniensis* on paired studies after 10 weeks; Table S2: Response to interaction observed upon gross mycelial contact between *P. leprieurii* var. *yasuniensis* and antagonists fungi after 28 weeks of growth; Table S3: Colonization of woodblocks and rhizomorph’s development in agar and soil microcosms at 30, 60, and 90 days; Table S4: Percentage of mass loss on agar and soil microcosms for 11 tropical woods; Table S5: Percentage of mass loss on agar and soil microcosms for 11 tropical woods inoculated with *P. leprieurii* var. *yasuniensis* after 30, 60, and 90 days; Table S6: Summary of “mother trees” in each site and the changes registered on the main substrate and other available substrates.
