# Supplementary material for: New Findings on the Biology and Ecology of the Ecuadorian Amazon Fungus Polyporus leprieurii var. yasuniensis
Source: J Fungi (Basel). 2022 Feb 20;8(2):203. doi: 10.3390/jof8020203 (PMC8874993; doi:10.3390/jof8020203)
Supplement: Supplementary file 1 [file jof-08-00203-s001.zip › jof-1582422-supplementary.pdf]

Supplementary materials

Table S1. Radial growth in mm (mean ± standard deviation) and percentage of growth inhibition (mean ± standard deviation) of *P. leprieurii* var. *yasuniensis* (CT-16F) on paired studies with other fungi after 10 weeks.

	Radial growth mm		% Inhibition growth	
**Pair**	**MYA**	**MEA**	**MYA**	**MEA**
A*	75.00	69.58		
A-1	58.07 ± 10.20	49.79 ± 3.74	22.57 ± 13.60	27.92 ± 9.44
A-2	44.74 ± 5.80	47.28 ± 2.55	40.34 ± 7.73	31.73 ± 6.08
A-3	20.37 ± 1.94	16.22 ± 1.74	72.83 ± 2.58	76.50 ± 3.60
A-4	75.00 ± 0.00	32.82 ± 6.01	0.00 ± 0.00	52.48 ± 9.88
A-5	35.02 7.33	20.35 ± 1.16	53.30 ± 9.77	70.67 ± 1.70
A-6	17.22 ± 2.41	13.64 ± 1.45	77.05 ± 3.22	80.33 ± 2.21
A-7	18.48 ± 0.98	13.18 ± 1.83	75.35 ± 1.30	81.03 ± 2.45
A-8	33.70 ± 14.82	26.12 ± 6.68	55.06 ± 19.76	62.68 ± 7.80
A-9	75.00 ± 0.00	57.18 ± 18.77	0.00 ± 0.00	18.33 ± 23.27
A-10	17.74 ± 3.88	16.33 ± 1.39	76.35 ± 5.17	76.52 ± 1.28
A-11	42.56 ± 7.64	34.20 ± 1.34	43.25 ± 10.18	50.60 ± 4.42
A-12	75.00 ± 0.00	40.47 ± 10.83	0.00 ± 0.00	41.14 ± 18.46
A-13	43.25 ± 8.92	30.59 ±3.80	42.33 ± 11.90	56.06 ± 4.13
A-14	33.92 ± 4.52	28.41 ± 3.22	54.78 ± 6.03	59.10 ± 4.29
A-15	67.85 ± 10.91	37.11 ± 7.80	9.53 ± 14.54	46.93 ± 8.07
A-17	33.02 ± 8.69	45.72 ± 5.45	55.98 ± 11.59	33.53 ± 12.76
A-18	35.90 ± 3.79	23.66 ± 0.98	52.13 ± 5.06	65.78 ± 3.70

*Control.

Table S2. Response to interaction observed upon gross mycelial contact between *P. leprieurii* var. *yasuniensis* and antagonistic fungi after 28 weeks of growth.

	Response to interaction		Success	
**Pair**	**MYA**	**MEA**	**MYA**	**MEA**
A*	A	A, B		
A-1	A, B, C, F	A, B	A	A
A-2	A, B, C	A, B,	A	A
A-3	A, B. C, D, F	C, D, F	3	3
A-4	A, B. C, F	A, B, C, D	A	4
A-5	A, B, C, D, E, F	A, B	5	5
A-6	C, D, F	C, D, F	6	6
A-7	C, D, F	A, C, D, F	7	7
A-8	A, B, C, D, F	A, B, C	A-8	A
A-9	A, B, C, D, F	A, B, C	A	A

A-10	A, B, C, D, F	A, B, C, D, F	A-10	A
A-11	A, B, C	A, B	A	A-11
A-12	A, B, C, F	A, B, C, E, F	A	A12
A-13	A, B, C	A, B	A13	A13
A-14	A, B, C, D, E, F	C, D, E, F	14	14
A-15	A, B, C, F	A, B, C	A	A
A-17	A, B, C, D, F	A, B, C, F	A-17	A-17
A-18	A, B, C, D	A, C, E, F	A-18	A-18

*Controls, *P. leprieurii* var. *yasuniensis* isolate CT-16F alone. **a.** pigmentation; **b.** zone lines (pseudosclerotial plates); **c.** overgrowth; **d.** aerial tufts; **e.** mycelial cords; **f.** replacement (relic zone lines) and success of *P. leprieurii* var. *yasuniensis* or antagonist at 28 weeks.

Table S3. Colonization of woodblocks and rhizomorph development in agar and soil microcosms at 30, 60, and 90 days.

	**Woodblock's colonization and Rhizomorph's development**					
	30 day		60 days		90 days	
**Wood**	**Agar**	**Soil**	**Agar**	**Soil**	**Agar**	**Soil**
Balsa	T/N *	T/Y	T/N	T/Y	T/Y	T/Y
Pachaco	T/N	T/Y	T/N	T/Y	T/N	T/Y
Melina	P/N	T/Y	P/N	T/Y	T/N	T/Y
Guayacan	P/N	T/Y	P/N	T/Y	P/N	T/Y
Laurel	T/N	T/Y	T/N	T/Y	T/N	T/Y
Cedro	P/N	T/Y	P/N	T/Y	P/N	T/Y
Seike	P/N	T/Y	P/N	T/Y	P/N	T/Y
Teka	P/N	T/N	P/N	T/N	P/N	T/Y
Colorado	T/N	T/Y	T/N	T/Y	T/N	T/Y
Saman	P/N	P/Y	P/N	T/Y	P/N	T/Y
Moral	P/N	P/N	P/N	P/N	P/N	P/Y

* T = Total colonization; P = Partial colonization/Y = yes development of rhizomorphs; N = no development of rhizomorphs.

Table S4. Percent mass loss of 11 tropical woods used as controls and noninoculated on agar and soil microcosms after 30, 60, and 90 days.

	**Agar**			**Soil**		
**Wood**	**30 day**	**60 days**	**90 days**	**30 day**	**60 days**	**90 days**
Balsa	−0.28 ± 0.38	0.63 ± 0.45	0.06 ± 0.29	0.28 ± 0.39	0.07 ± 0.16	−0.7 ± 0.66
Pachaco	0.18 ± 0.15	−0.02 ± 0.12	0.08 ± 0.09	0.12 ± 0.16	0.05 ± 0.07	0.43 ± 0.04
Melina	0.09 ± 0.10	0.12 ± 0.13	0.11 ± 0.10	0.11 ± 0.15	0.00 ± 0.00	0.23 ± 0.06
Guayacan	0.01 ± 0.04	0.12 ± 0.03	0.00 ± 0.00	0.00 ± 0.00	0.00 ± 0.00	0.13 ± 0.07
Laurel	0.07 ± 0.10	0.17 ± 0.10	−0.02 ± 0.05	0.16 ± 0.17	0.08 ± 0.11	0.24 ± 0.16

Cedro	0.06 ± 0.06	0.11 ± 0.19	0.05 ± 0.08	0.09 ± 0.13	0.06 ± 0.13	0.06 ± 0.14
Seike	−0.03 ± 0.11	0.08 ± 0.10	−0.002 ± 0.04	0.11 ± 0.14	−0.02 ± 0.04	0.06 ± 0.16
Teka	0.08 ± 0.08	0.07 ± 0.11	0.00 ± 0.00	0.11 ± 0.07	0.05 ± 0.10	0.16 ± 0.12
Colorado	0.13 ± 0.08	0.04 ± 0.11	0.00 ± 0.00	0.02 ± 0.03	0.03 ± 0.15	0.12 ± 0.11
Saman	0.03 ± 0.12	0.03 ± 0.12	0.01 ± 0.20	0.003 ± 0.21	0.06 ± 0.22	0.14 ± 0.10
Moral	0.89 ± 0.09	1.08 ± 0.12	0.01 ± 0.01	1.14 ± 0.40	−0.06 ± 0.60	1.30 ± 0.14

Table S5. Percent mass loss for 11 tropical woods (mean ± standard deviation) inoculated with *P. leprieurii* var. *yasuniensis* after 30, 60, and 90 days on agar and soil microcosms.

	**Agar**			**Soil**		
**Wood**	**30 day**	**60 days**	**90 days**	**30 day**	**60 days**	**90 days**
Balsa	7.72 ± 3.48	15.01 ± 5.36	22.73 ± 3.96	11.02 ± 3.17	12.13± 3.68	17.04 ± 2.05
Pachaco	4.08 ± 0.89	10.72 ± 1.65	16.74 ± 1.98	9.33 ± 0.86	14.20 ± 7.55	29.45 ± 6.14
Melina	1.27 ± 0.72	6.26 ± 4.72	13.98 ± 4.71	6.91 ± 2.02	12.75 ± 4.45	20.15 ± 3.70
Guayacan	1.52 ± 1.57	2.17 ± 1.39	3.90 ± 3.17	4.89 ± 0.87	10.07 ± 2.20	20.64 ± 1.98
Laurel	1.87 ± 0.72	7.31 ± 0.99	11.96 ± 1.51	4.68 ± 0.96	10.15 ± 1.74	14.78 ± 1.93
Cedro	0.79 ± 0.09	1.06 ± 0.21	3.5 ± 2.48	2.57 ± 0.40	4.61 ± 1.88	10.64 ± 5.38
Seike	0.90 ± 0.16	1.47 ± 1.13	1.48 ± 0.29	2.71 ± 0.47	4.15 ± 0.33	7.81 ± 1.06
Teka	0.56 ± 0.33	1.73 ± 0.46	1.44 ± 0.76	2.08 ± 1.34	2.89 ± 0.84	7.56 ± 2.63
Colorado	1.06 ± 0.12	1.85 ± 0.35	3.36 ± 0.32	1.53 ± 0.28	2.97 ± 0.14	6.28 ± 1.25
Saman	1.06 ± 0.21	1.83 ± 0.55	3.97 ± 1.72	0.98 ± 0.29	1.83 ± 0.95	5.29 ± 0.96
Moral	0.71 ± 0.10	1.23 ± 0.12	1.45 ± 0.29	1.50 ± 0.23	1.74 ± 0.33	3.50 ± 1.78

Table S6. Summary of "mother trees" at each site and the changes registered on the main substrate and other available substrates. Also, a description of rhizomorphs and basidiocarps found at the beginning and end of observations.

**Site**	**Tag #**	**Tree species**	**Initial stage**	**Final stage**
CT-16F	164893	*Plinia* sp.	Standing dead with basidiocarps and rhizomorphs emerging from the roots and root collar. Sclerotial plates are present where rhizomorphs and basidiocarps emerge. In the area, rhizomorphs and basidiocarps emerge from small branches found on the ground.	Fallen on the ground with symptoms of butt-rot (main stem broken at the root collar) and advanced decay. Base and main stem covered with rhizomorphs and basidiocarps. Rhizomorphs and basidiocarps are abundant on-site, emerging from coarse and fine-woody debris that are widely distributed around the base of "mother tree" and downhill.

CTR-2-12	215267	*Quiina* sp.	Fallen suspended dead tree with butt-rot symptoms, as it is broken at the root collar and exhibits white rot. The tree is fallen downhill. Rhizomorphs and basidiocarps emerge from the main stem and branches that are dispersed in the area.	The tree has been broken into three sections, some remain suspended and others on the ground. Advanced decay is observed on small pieces of wood, while the main stem is still solid. Abundant rhizomorphs emerge from the main stem and basidiocarps from small branches that are dispersed on the ground. Abundant rhizomorphs and basidiocarps are present in the area emerging from coarse and fine-woody debris, colonizing seedlings and trees with tags.
CTR-2-27	410664	*Virola duckei*	Fallen dead tree with symptoms of butt-rot (broken at the root collar level) and advanced decay on the base. Rhizomorphs and basidiocarps emerge from the main stem but are not present on the base. Coarse and fine-woody debris with rhizomorphs and basidiocarps are dispersed in the area.	Advanced decay in the main stem, which has almost completely decayed. Rhizomorphs and basidiocarps remain abundant on the site emerging from coarse and fine-woody debris, but only a few emerge from the main stem. Rhizomorphs and basidiocarps remain abundant on-site emerging from coarse and fine-woody debris distributed on the ground around "mother tree".
CTR-2-31	420636	*Pseudolmedia rigida*	Fallen dead tree with symptoms of butt-rot (broken at the root collar level) and white rot, however wood is still solid. Fallen downhill. Abundant rhizomorphs and basidiocarps emerge from the main stem and small branches that are dispersed in the area.	The tree remains on the ground, apparently still in early stages of decay as wood feels solid, but with most of the bark gone and abundant rhizomorphs and basidiocarps emerging from the main stem. Rhizomorphs and basidiocarps remain abundant, emerging from coarse and fine-woody debris that extend to colonize seedlings and trees with tags.
CTR-2-67	No tag number	Not known	The area is partially flooded with abundant coarse and fine-woody debris dispersed on the ground. Rhizomorphs and basidiocarps emerge from woody debris that are submerged, while rhizomorphs stand upright above the water, some attached to trees and other plant material.	Similar observations regarding substrate availability and flooding conditions. More colonization is observed in logs of 15 cm diameter and of 0.5-1.5 meters long with rhizomorphs emerging from woody debris to adhere to the new substrates. The site seems to be abundantly colonized by rhizomorphs and basidiocarps found to be colonizing seedlings and a few trees with tags.
